# Analysis of risk factors progression of preterm delivery using electronic health records

**DOI:** 10.1186/s13040-022-00298-7

**Published:** 2022-08-17

**Authors:** Zeineb Safi, Neethu Venugopal, Haytham Ali, Michel Makhlouf, Faisal Farooq, Sabri Boughorbel

**Affiliations:** 1 Research Department, Sidra Medicine, Doha, Qatar; 2 Division of Neonatalogy, Sidra Medicine, Doha, Qatar; 3 Department of Maternal-Fetal Medicine, Sidra Medicine, Doha, Qatar; 4 Qatar Computing Research Institute, HBKU, Doha, Qatar

**Keywords:** Preterm, Pregnancy, EHR, Epidemiology, Risk factors, Progression, Temporal analysis, Precision medicine, Predictive models

## Abstract

**Background:**

Preterm deliveries have many negative health implications on both mother and child. Identifying the population level factors that increase the risk of preterm deliveries is an important step in the direction of mitigating the impact and reducing the frequency of occurrence of preterm deliveries. The purpose of this work is to identify preterm delivery risk factors and their progression throughout the pregnancy from a large collection of Electronic Health Records (EHR).

**Results:**

The study cohort includes about 60,000 deliveries in the USA with the complete medical history from EHR for diagnoses, medications and procedures. We propose a temporal analysis of risk factors by estimating and comparing risk ratios and variable importance at different time points prior to the delivery event. We selected the following time points before delivery: 0, 12 and 24 week(s) of gestation. We did so by conducting a retrospective cohort study of patient history for a selected set of mothers who delivered preterm and a control group of mothers that delivered full-term. We analyzed the extracted data using logistic regression and random forests models. The results of our analyses showed that the highest risk ratio and variable importance corresponds to history of previous preterm delivery. Other risk factors were identified, some of which are consistent with those that are reported in the literature, others need further investigation.

**Conclusions:**

The comparative analysis of the risk factors at different time points showed that risk factors in the early pregnancy related to patient history and chronic condition, while the risk factors in late pregnancy are specific to the current pregnancy. Our analysis unifies several previously reported studies on preterm risk factors. It also gives important insights on the changes of risk factors in the course of pregnancy. The code used for data analysis will be made available on github.

## Introduction

The WHO defines prematurity as births before 37 weeks of completed gestation [1]. Over the recent years there is a global increase in the rate of prematurity, ranging from 9-12% of all birth. In 2016, prematurity affected around 15 million live-born babies worldwide. [2] The reporting of the short and long-term outcomes of prematurity attracted the interest of scientists, clinicians, as well as policymakers. Of particular concern is the high rate of prematurity within the low socioeconomic class [3]. Extreme prematurity, defined as birth before 28 weeks of gestation, poses life-long consequences on the health, education, and the social life of children [4]. From the societal perspective, almost one-third of extremely premature infants required support in special education systems. The majority had poor educational attainment at school age [5]. It was found that 10% of the deaths were due to preterm birth among the six major causes that were attributed to 73% of yearly deaths of children under the age of 5 years [6]. Children that are born preterm are at a higher risk of a plethora of psychological and physiological health implications, especially those with a low birth weight. A quarter of the very low birth weight children develop severe or multiple psychological problems, and another quarter develop moderate to mild problems. The psychological development is measured in different domains, namely cognitive development, behavioral and emotional status, social functioning and school adaptation [7]. Children that are very preterm have abnormal brain morphology when compared to those born full-term at seven years of age [8]. A Significant proportion of the children that are born very premature, i.e., before 27 week gestation, are found to have difficulties in motor and academic skills in early school years [9]. They are also more likely to develop respiratory problems [10]. Preterm deliveries have many negative implications on the mother’s and child’s health alike. Mothers that deliver preterm babies, are at a greater risk to suffer from complications, particularly those who undergo a Cesarean delivery. The complications include hemorrhage, infection, ICU admission and death [11].

Identifying mothers that are at a higher risk through quantifying risk factors of preterm delivery at a population level helps clinicians to take preventive measures and mitigate the risks [12, 13]. Traditionally, the identification of such risk factors is done through prospective studies. This method presents challenges, some of which are difficulty in recruiting trial participants and the tediousness of the data collection process. With the increased adoption of Electronic Health Records (EHR) in hospitals and health care facilities around the world, some of these challenges have been alleviated. EHR data is being extensively used in clinical research, even though it presents some challenges of its own, it provides fast and easy access to large amounts of data, that is more representative of the general population than data collected from clinical trials. Some of the research application of EHR data are epidemiology and observational research, safety surveillance and regulatory use, and prospective clinical research [14].

Most studies are also concerned with extracting risk factors from a limited set of suspected attributes, while in our analyses we extract risk factors from all possible attributes that are available to us in Electronic Health Records. We included diagnosis information (ICD-9 and ICD-10), medication information (NDC codes), laboratory orders (ICD-9 and ICD-10).

The purpose of this work is to perform a retrospective cohort study using a large EHR dataset to identify risk factors of preterm delivery. We hypothesize that risk factors are dynamics and can change in the course of pregnancy. In the early phase of pregnancy, genetic predisposition might dominate the risk factors while in advanced stages of the pregnancy, other factors could play more important roles [12]. The analysis of the variation of risk factors as a function of time could help in building models for the early prediction of preterm delivery.

In this work, we identify the risk factors that are associated with preterm delivery in the literature, and present a few examples of the use of EHR data to perform epidemiology studies in “Related work” section. We present the methodology adopted for extracting relevant preterm and full-term deliveries from the EHR database and statistical analysis of the data in “Methodology” section. We present our results and discuss our findings in “Results and discussion” section. Risk factors obtained from association studies should not be understood as causal factors. Among the risk factors that are statistically significant, we curated the ones related to spontaneous preterm deliveries from the medically induced ones. “Conclusion” section concludes our findings and presents prospects for future work.

## Related work

### Preterm delivery risk factors

The identification of risk factors of preterm deliveries is a well researched problem. The history of previous preterm deliveries is one of the main risk factors that have been identified by different research studies [15–18]. Maternal age is also a risk factor, adolescent pregnancies and advanced maternal age are associated with increased risk of preterm deliveries [15, 16]. Other factors are low maternal BMI [15, 18], obesity [16], women of African race [16–18], and short inter-pregnancy intervals [16, 17]. The mother anatomy, such as short cervical length [16–18], and uterine anomalies [17] are also well known risk factors. Existing conditions in the mother whether they are infectious conditions (HIV, chlamydia, and urinary tract infection) or other conditions (pre-eclampsia, low maternal vitamin D, pregestational and gestational diabetes [16], and anemia [19]) also increase the risk of preterm delivery. Another risk factor is multiple gestation [18] or singleton and twin pregnancies formed through in vitro fertilization [16], in addition to some of the mother’s behaviors such as smoking and drug use during pregnancy [16]. Some work also investigated the role of environmental factors on preterm birth [20], where an increase in *NO*_2_ concentration was shown to have an association with preterm birth.

### Risk factors from electronic health records

With the increasing worldwide adoption of Electronic Health Records, many research groups made use of the availability of such large data volumes for different medical research purposes. Epidemiology studies are use cases that are becoming increasingly popular. An example application is finding associations between late-preterm birth and persistent asthma in young children, inhaled corticosteroid use and more acute respiratory visits, by conducting a retrospective cohort analyses [10]. EHR data was also used to examine the association between the exposure to psychotherapy during pregnancy and the risk reduction of adverse pregnancy outcomes among women with history of major depressive disorder and obstetric outcomes [21]. Other examples include associating glucocorticoid use to predicting fracture risk [22], using EHR data to predict the epidemiology of disease biomarkers [23], and identifying the risk factors of Angiotensin -converting enzyme inhibitors (ACEIs) by conducting a retrospective cohort study from EHR data [24].

## Methodology

**Fig. 1:**
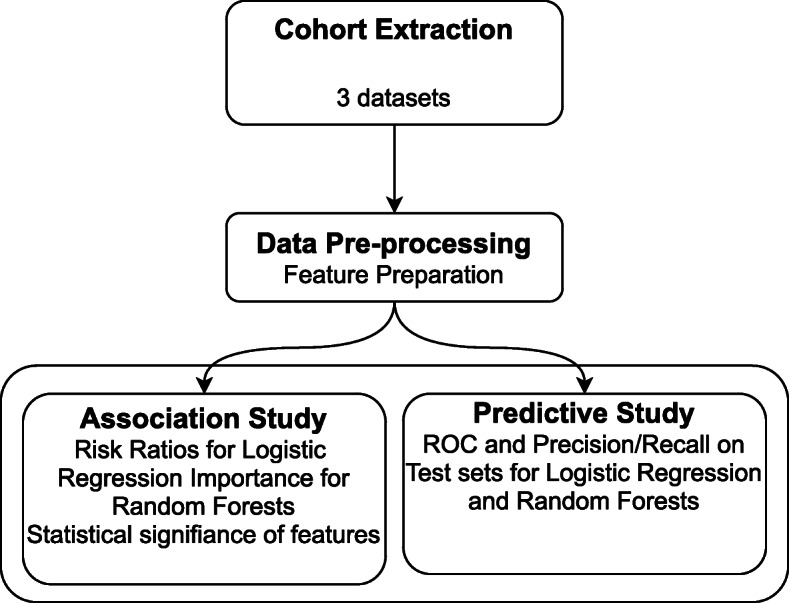
Workflow of the data analysis

**Fig. 2:**
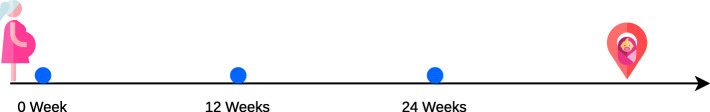
Timeline of the clinical history for different pregnancies included in the cohort. Each line in the figure depicts a mother’s history timeline. The dots on the lines represent delivery events. Green dots represent full-term deliveries, while red dots represent preterm deliveries. The post delivery visits were excluded from the data in the analyses. The dark gray portion before the delivery event is the time gap between the analysis time point and delivery. The data in this time interval were also omitted from the mother’s history

The analysis plan is illustrated in Fig. 1. We considered association analysis for risk factor identification and predictive analysis for preterm-birth prediction. In order to identify the variation of risk factors in the course of pregnancy, we performed association studies at three time points during pregnancy. These time points are 0, 12 and 24 gestational weeks. At each time point, only the past medical history with respect to the time point is included for each subject. For example, if the time point of analysis is 12 gestational weeks, all the data between 12 gestational weeks and the delivery event are discarded. This mimics a realistic scenario where future data is not available at each time point of analysis. Figures 2 and 5 illustrate the data timeline used in the analysis. The gestational age are extracted based on ICD-10 codes as described in the following section. The inclusion of the time dimension in the analysis gives us a predictive tool. The risk factors at different time points can be interpreted as risk predictors for preterm delivery. The association study is based on multivariate logistic regression model.

### Data extraction

**Fig. 3:**
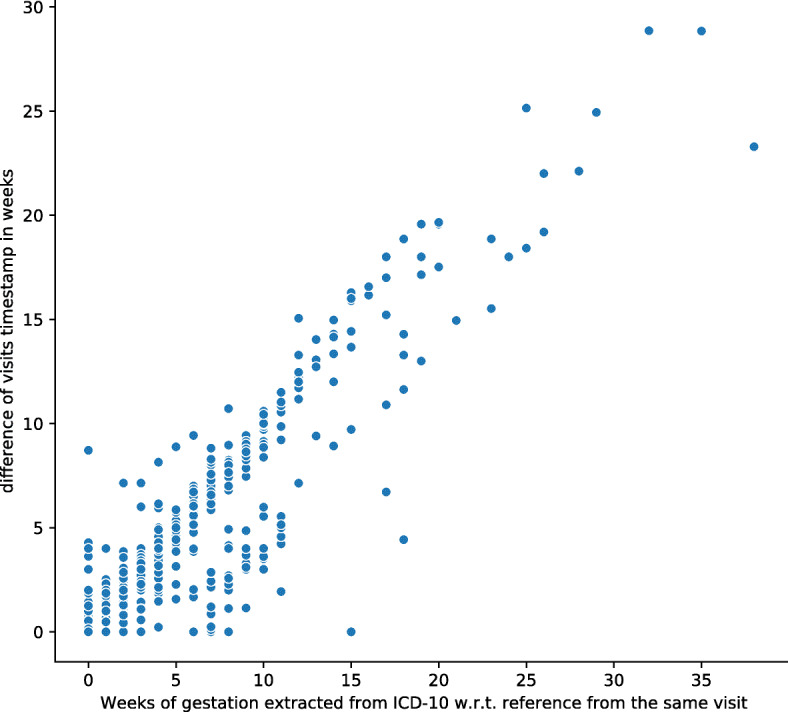
Time difference of EHR visits based on extracted gestational age from ICD-10 visit codes

**Fig. 4:**
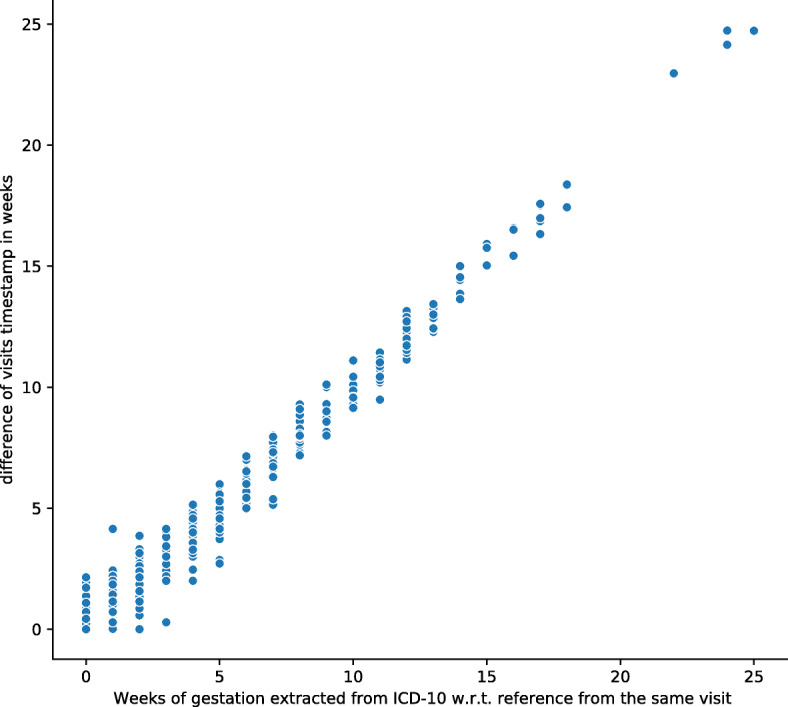
Similar plot as in Fig. 3 with a filtered visits based on RMSE ≤1 week between the time difference from extracted gestational ages and visit timestamps

We used Health Facts^Ⓒ^ Cerner EHR database to extract the dataset that is used in our analyses. The dataset consists of a retrospective and longitudinal cohort of full-term and preterm deliveries in the period 2001-2017. The dataset of our cohort is gathered from 120 hospitals in the USA. Gestational ages of the pregnancies were extracted using Z3A.xx codes which indicate weeks of gestation. For example Z3A.21 specifies 21 weeks gestation of pregnancy. We selected pregnancies with at least two ICD-10 codes of gestational age. We validated these codes based on the visit time stamps. We checked if the duration using EHR time stamps between the visits having gestational codes is consistent with the duration based on gestational ages extracted from the ICD-10 codes. For example Let’s assume a pregnancy had two visits with ICD-10 codes of Z3A.25 (March 1) and the Z3A.38 (June 20). The difference based on gestational age is 13 weeks (91 days) while the difference between the visit time stamps is 110 days. This difference could be due to errors or delay in reporting the information in the EHR system. We discarded pregnancies if the error is higher than one week. Figure 3 shows a validation of the extracted gestational ages. Our assumption is that if the gestational ages extracted from the diagnosis codes are accurate then the time elapsed between two visits should be the same if calculated as the difference of gestational ages or as the difference of time stamps of the visits from EHR. For each subject we took the first visit as a reference point and subtracted the time stamps (x-axis) from the reference as well as the gestational ages (y-axis). Each point in the figure corresponds to information about one visit. In order to clean the data from erroneous gestational age we removed subjects having a large deviation in terms of time difference based on gestational age and timestamps. We discarded samples with a Root Mean Square Error (RMSE) larger than one week. Figure 4 shows an example of samples after removing the potential outliers.

We relied on ICD-10 diagnosis codes to identify preterm and full-term pregnancies. The ICD-10 codes that was used to identify preterm and full deliveries are provided in the supplementary material. In order to validate the data extraction, we randomly selected 100 samples and presented them to a clinician for a manual check. He concluded that for 11 records among 100 it was not possible to assert whether the delivery was full or preterm. For the remaining records, the associated labels (full term delivery vs. preterm delivery) were accurate. We note the clinician decision was based on the information available from EHR which did not include clinical notes.

**Fig. 5:**
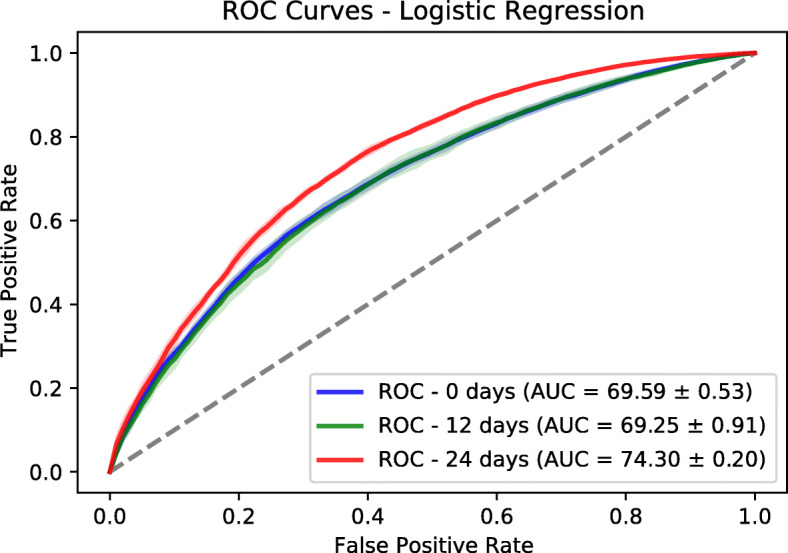
Time points used to predict preterm-birth

To evaluate the risk factors at different time intervals prior to delivery event, we excluded patient history from the delivery event until a defined time point. Figure 2 illustrates the data selection process. We first used a time gap of 0 week of gestation, and selected mothers that had at least two hospital visits in their history prior to the time gap. Mother E in Fig. 2 would not be selected in this case, as not enough history data is available for the analyses. These time points are illustrated in Fig. 5.

The medical history of each patient encompasses the following information: drug orders (using NDC encoding), procedures (using ICD-9 ICD-10, CPT4, and HCPCS encoding), diagnosis codes (using ICD-9 and ICD-10 encoding) and lab orders (using LOINC encoding). In addition to these, the dataset includes demographic information of the patients. The latter included are race, marital status, age, medical specialty and hospital ID. The demographic information are included to reduce the effects of confounding factors.

### Risk factors identification

We used logistic regression as shown in Eq. (1) [25] for risk factor identification using four datasets, each containing the same set of patients, with a portion of the data omitted according to the previously mentioned time gaps.

1 $$  \pi(x) = \frac{e^{\beta_{0} + \beta_{1} x_{1} + \beta_{2} x_{2} +... + \beta_{p} x_{p}}}{1+e^{\beta_{0} + \beta_{1} x_{1} + \beta_{2} x_{2} +... + \beta_{p} x_{p}}}  $$

In Eq. (1), *β*_*i*_ are the coefficients to be estimated and *x*_*i*_ values are the corresponding independent variables that represent medications, diagnosis, lab orders, procedures and demographics. The value of i ranges from 1 to p, where p is the total number of dependent variables.

The dependent variable in the analysis *y* indicates whether the delivery is preterm *y*_*i*_=1 or full-term *y*_*i*_=0. We used Python’s scikit-learn^1^ implementation of logistic regression. We used an SGD training with *L*_2_ regularization (*α*=0.01) and tolerance=0.001, batch size of 5000 and 30 epochs.

After estimating the multiple logistic regression coefficients, we extracted, from the multivariate model, the *p*-values associated with each predictor. This allowed us to evaluate the statistically significant variables in the analysis. A Bonferroni correction was applied to these *p*-values. The covariates with the significant adjusted *p*-values were selected as risk factors in the corresponding time gap. The *p*-value cut-off was chosen to be 0.01 to retain the statistical significant variables. We report the risk ratios as well as their 95% confidence intervals corresponding to each identified statistically significant risk factor.

The rate of preterm birth is reported to be 1:10 in the US according to CDC 2. In our dataset the preterm birth rate is about 9.5%, which is very close to the reported risk ratios. In order to account for the potential mismatch between the observed and the real prevalence of preterm birth we use Eq. (2) to calculate the corrected risk ratio, where OR is the odds ratio and *P*_0_ is the incidence of the covariate in the full-term group [26].

2 $$  RR = \frac{OR}{(1-P_{0})+(P_{0} \times OR)}  $$

**Fig. 6:**
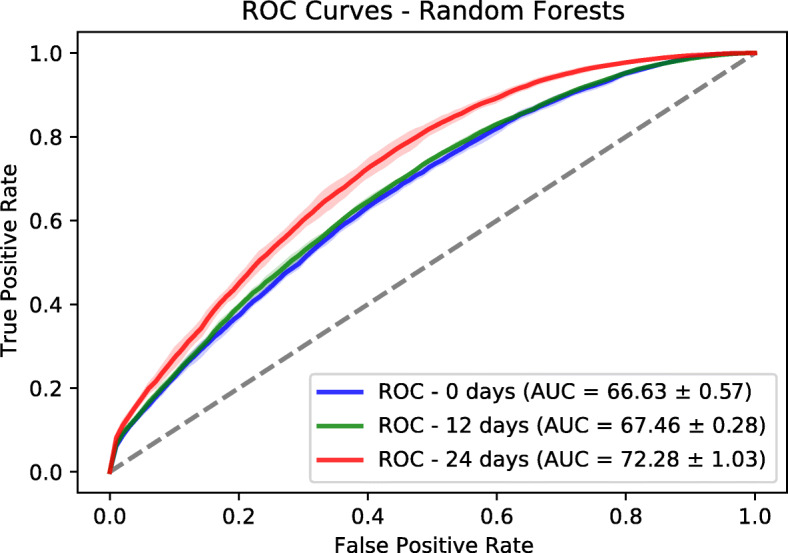
ROCs on test sets based on Logistic Regression

**Fig. 7:**
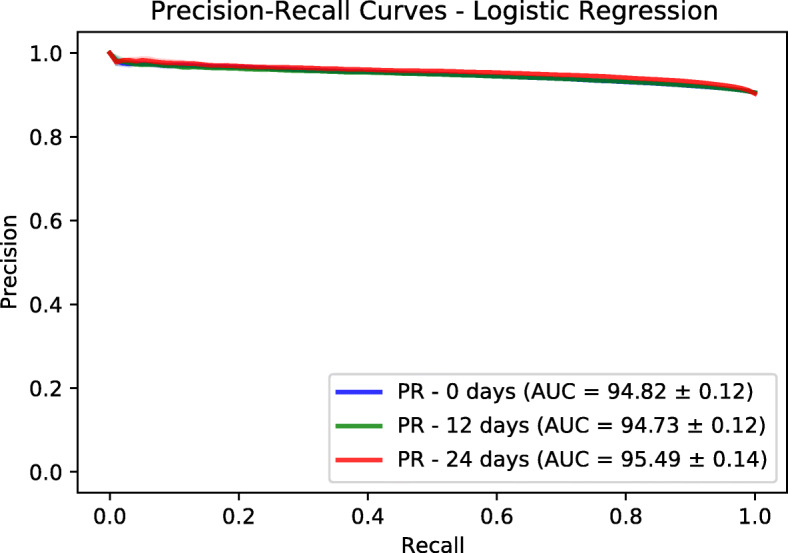
ROCs on test sets based on Random Forests

**Fig. 8:**
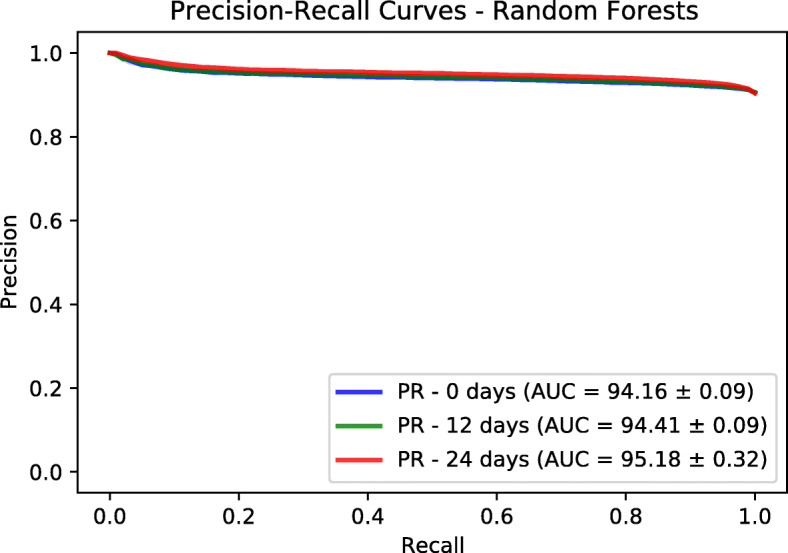
Precision-Recall curves on test sets based on Logistic Regression

**Fig. 9:**
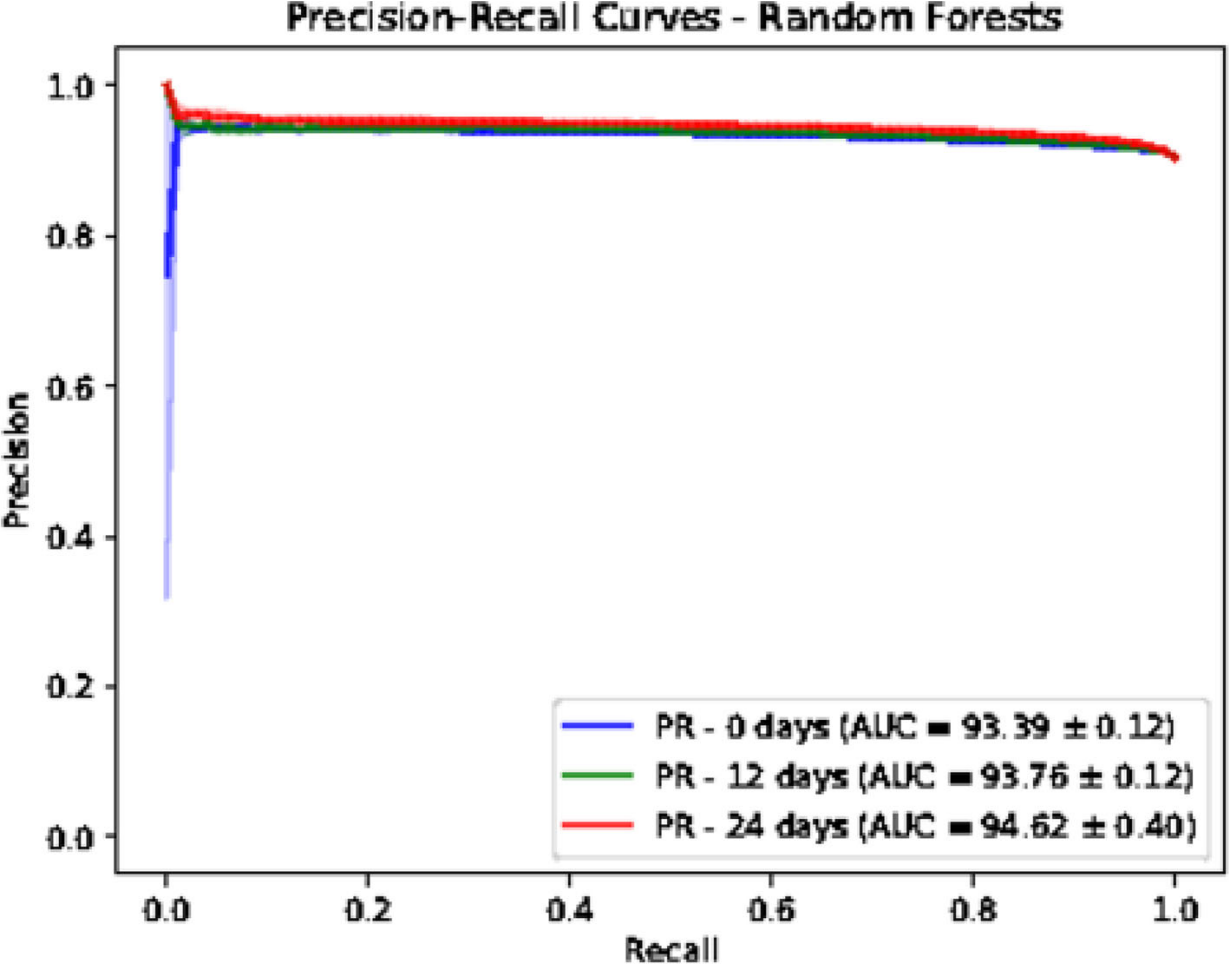
Precision-Recall curves on test sets based on Random Forests

In addition to Logistic Regression model, we trained a Random Forests model based on a fast implementation in Ranger package [27]. The number of trees is set to 500. The number to possibly split in each node is 185. The regularization factor (gain penalization) is 1 [28]. These are the default values in Ranger package. We extracted the variable importance of each feature and the associated *p*-value based on an approximation of a permutation test [29]. We performed a Bonferroni *p*-value correction and we used the value of 0.01 to identify statistically significant features. We used a threshold of 3 on the variable importance to retain the most relevant features. We evaluated the predictor power of both logistic regression and random forests using a held-out test sets corresponding to 30% of the dataset size. The splitting and evaluation was repeated 10 times for each model. We used area under ROC and Precision-Recall curves as performance metrics. We provide mean and standard deviation for each metric. The results are reported in Figs. 6, 7, 8 and 9. Logistic regression performed better than random forests on the prediction of preterm-birth. Therefore we present the results of logistic regression in the main text and provide Random forests variable importance in the supplementary material. The code used for data analysis will be made available on github^3^.

## Results and discussion

### Datasets

The datasets that were used in the experiment contained about of 60,000 delivery events with a prevalence of about 9.5% of preterm deliveries. The size of the datasets are reported in Table 1. The covariates in the experiments are the different medications, procedures, lab orders, and diagnosis that occurred more than 100 times across all patient visits at each time gap. The number of covariates changes across the datasets, as some of them that appeared in a specific time gap from the delivery would disappear as the time gap between the delivery event and the start of considered history is made bigger. A summary description of the covariates for the different clinical modalities is given in Table 1.

**Table 1:** Covariate Counts by Category

Category	0 Week	12 Weeks	24 Weeks
Diagnosis	17250	17169	17266
Medication	12892	12506	12287
Procedure	6282	6110	4596
Lab orders	2878	2811	2770
Total	39302	38596	36919
# samples	63814	60431	58696
Preterm %	9.5	9.4	9.6

### Models evaluation

To evaluate the goodness-of-fit of the logistic regression and random forest models we used the Area Under the receiver operating characteristic Curve (AUC) and the Area under the Precision and Recall Curve (PRC) of the different datasets. We tuned the hyper-parameters of the Logistic Regression (LR) and Random Forests (RF) on the training dataset using a grid search approach. For LR, we tuned the type of regularization [ *L*_1, *L*_2] and the regularization factor with possible values as [0.001, 0.01, 0.1, 1]. The choice of *L*_2 and factor of 0.01 gave the best performance across the three prediction time points. The different AUC values are shown in Table 2. For RF, we tuned two hyper-parameters which are the number of trees (ntree) and the number of variables to possibly split at in each node (mtry). The possibles values are [10, 100, 500] for ntree and [10, 50, 250] for mtry. The choice of ntree=10 and mtry=250 was the best from the grid search.

**Table 2:** Prediction performance for Logistic Regression (LR) and Random Forests (RF) models

Number of weeks	0	12	24
AUC - LR	69.59 ±0.53%	69.25 ±0.91%	74.30 ±0.20%
AUC - RF	66.63 ±0.57%	67.46 ±0.28%	72.28 ±1.03%
PRC - LR	94.82 ±0.12%	94.73 ±0.12%	95.49 ±0.14%
PRC - RF	94.16 ±0.09%	94.41 ±0.09%	95.18 ±0.32%

### Identified risk factors based on logistic regression

Tables 3 and 4 summarize the significant risk factors identified. The risk factors that are identified in each time gap are those with a *p*-value ≤0.01. To follow the progression of risk factors before and during the pregnancy at different stages, we group the risk factors that are common to all three time gaps and two time gaps (Table 3), as well as those that are specific to the 24 gestation-weeks time gap, 12 gestation-weeks time gap and 0 gestation-week time gap (Table 4). The tables show the medication, diagnosis, medical procedure and lab orders codes proceeded by M_, D_, P_ and L_ respectively in the first columns of the table. The corresponding descriptions of these codes are also included in the table. The description of medication codes consist of brand names and generic names of the medication separated by “/”. Risk factors with a risk ratio between 0.9 and 1.1 are excluded from the analysis, as the coefficients of these risk ratios are very close to 1, and are found to not have a significant impact.

**Table 3:** Common risk factor from Logistic Regression across all time gaps

	Code	Description	Risk Ratios(95% CI)		
			0 Week	12 Weeks	24 Weeks
All time gaps	D_644.21	Early Onset of Delivery, Delivered, with or without Mention of Antepartum Condition	2.03(1.96-2.1)	1.93(1.82-2.05)	1.78(1.6-1.95)
	M_85056605.0	Celestone Soluspan	1.76(1.66-1.85)	2.05(1.93-2.16)	1.81(1.71-1.92)
	M_517072001.0	Betamethasone Acetate-Betamethasone Sodium Phosphate	1.74(1.66-1.83)	1.92(1.82-2.03)	1.76(1.7-1.83)
2 time gaps	M_409672909.0	Magnesium Sulfate, Injectable/magnesium sulfate	1.68(1.49-1.87)		1.78(1.58-1.98)

**Table 4:** Risk factors from Logistic Regression unique to 0, 12 and 24 week(s)

	Code	Description	Risk Ratios(95% CI)
0 week	M_409672909.0	Magnesium Sulfate, Injectable/magnesium sulfate	1.68(1.49-1.87)
	L_26464-8	White Blood Cell Count, Blood	1.75(1.54-1.96)
	D_654.20	Previous Cesarean Section, Unspecified as to Episode of Care or Not Applicable	1.59(1.46-1.72)
	L_32766-8	Trichomonas vaginalis	1.57(1.43-1.71)
	M_409672903.0	Magnesium Sulfate, Injectable/magnesium sulfate	1.57(1.42-1.71)
	D_V12.09	Other Personal History of Infectious and Parasitic Disease	1.54(1.37-1.7)
	M_55390012110.0	Ondansetron Hydrochloride/ondansetron	1.51(1.34-1.68)
	D_V15.81	Personal History of Noncompliance with Medical Treatment, Presenting Hazards to Health	1.47(1.32-1.62)
	D_644.20	Early Onset of Delivery, Unspecified as to Episode of Care or Not Applicable	1.46(1.31-1.61)
	L_741-9	Differential Microcytes	1.41(1.28-1.53)
	L_10378-8	Polychromasia Qualitative Blood Light Microscopy	1.41(1.27-1.54)
	D_645.11	Post Term Pregnancy, Delivered, with or without Mention of Antepartum Condition	0.49(0.35-0.63)
12 weeks	D_O30.041	Twin pregnancy, dichorionic/diamniotic, first trimester	1.65(1.62-1.69)
	D_O34.219	Maternal care for unspecified type scar from previous cesarean delivery	1.57(1.39-1.75)
24 weeks	D_O09.91	Supervision of high risk pregnancy, unspecified, first trimester	1.7(1.53-1.86)
	M_51079092920.0	Labetolol Hydrochloride/labetalol	1.73(1.58-1.87)
	D_O30.009	Twin pregnancy unspecified number of placenta and unspecified number of amniotic sacs unspecified trimester	1.45(1.39-1.51)

#### Risk factors common to all time gaps

The risk factors that are common to all time gaps, meaning, they indicate a high risk pregnancy before and during the current pregnancy, are the existence of a previous preterm delivery and possible pregnancy complications of previous pregnancy with preterm delivery. The previous preterm delivery is indicated by the diagnosis code D_644.21, and the medication Celestone Soluspan (Betamethasone), which is a prenatal corticosteroid that is administered in high risk pregnancies in order to reduce the risk of preterm infants having lung problem [30]. We clarify that Betamethasone as mentioned in Table 3 should not be interpreted as a risk factor for preterm birth but it is rather a proxy indicating the presence of previous pregnancy complications in the clinical history treated using Betamethasone.

#### Risk factors common to two time gaps

There is one significant factor common to two time gaps which is presented in Table 3. The risk factor which is a medication (Magnesium Sulfate, Injectable) prescribed for the treatment of pregnancy complication with risk of preterm delivery. This risk factor is also a proxy indicator of a previous preterm.

#### Risk factors at a single time gap

The results summary can be found in Table 4. The risk factors are most likely not relevant to the current pregnancy. They reflect preexisting chronic conditions in the mother’s history and general predisposition to deliver on preterm. The factors can be mainly grouped into three categories: 1) previous delivery complications: (D_654.20, D_644.20, M_55390012110.0, M_409672903). Magnesium Sulfate (M_409672903) and Ondansetron (M_55390012110.0) are used for the management of pregnancy complications [31]. 2) Complications related to blood: (L_26464-, L_741-9, L_10378-8) such as anemia or iron deficiency and 3) infectious diseases (D_041.9, L_32766-8). Anemia has been previously correlated with the risk preterm delivery [32–34]. Our results indicate that Previous post-term deliveries (D_645.11) reduces the risk of preterm delivery.

For 12 and 24 weeks of gestation, the significant ICD-10 codes are related to current high risk pregnancy. Twin pregnancy (D_O30.041) is known to be a risk factor of preterm delivery [18]. We found that previous cesarean delivery D_O34.219 is associated with preterm delivery. The medication (M_51079092920.0/ Labetolol) is used to treat hypertension. This finding is supported by previous research that pre-existing hypertension increases the risk of preterm delivery [35].

### Identified risk factors based on random forest

Similarly to Logistic Regression, Random Forest was used to identify the most important predictors for preterm delivery at different time points. We used a threshold of 2 for variable importance and the same *p*-value of 0.01 for identifying the statistical significant variables. The common risk factors for the three and two (0 and 12 weeks) time gaps as depicted in Table 5 is one factor which is a previous preterm delivery. Betamethasone is a proxy for a previous pregnancy with complications treated using this medication. The variables for two-time gaps are related to previous preterm delivery and twin pregnancy. Table 6 lists the risk factors unique to the time gap of 0, 12 and 24 week(s) of gestation. Three factors have been identified: Type 1 diabetes, blood complication identified by a lab order and JC/BK virus. A previous study based on a large cohort has found an association of type 1 diabetes with an increased risk of preterm delivery [36]. Previous preterm delivery and hypertension condition are the main identified factors. As the pregnancy progresses acute conditions dominate the risk factors. For example, Oligohydramnios (D_O41.02X0) is a condition of reduced amniotic fluid around your baby during pregnancy. A previous study has shown the association between Oligohydramnios and preterm risk [37]. Also Coagulation disorder (Fibrinogen, L_3255-7) is a known risk factor for preterm delivery [38].

**Table 5:** Risk factors from Random Forest common to two and three time gaps

	Code	Description	Variable Importance		
			0 Week	12 Weeks	24 Weeks
3 time gaps	M_517072001.0	Betamethasone Acetate-Betamethasone /betamethasone	3.53	3.73	9.98
2 time gaps	D_644.21	Early Onset of Delivery, Delivered, with or without Mention of Antepartum Condition	4.566	4.11	
	D_V23.41	Pregnancy with History of Pre-Term Labor	2.19	2.42	
	D_O30.041	Twin pregnancy, dichorionic/diamniotic, first trimester	7.17	6.03

**Table 6:** Risk factors from Random Forest unique to 0, 12 and 24 week(s) of gestation

	Code	Description	Variable Importance
0 week	D_250.01	Diabetes Mellitus without Mention of Complication, Type I	2.64
	L_49024-3	Differential Cell Count Method Blood	2.09
	L_47251-4	JC/BK Virus PCR	2.07
12 weeks	D_401.9	Unspecified Essential Hypertension	2.13
	D_644.20	Early Onset of Delivery, Unspecified as to Episode of Care or Not Applicable	2.11
	D_O60.10X0	Preterm labor with preterm delivery, unspecified trimester, not applicable or unspecified	2.05
24 weeks	D_O30.032	Twin pregnancy, monochorionic/diamniotic, second trimester	7.62
	D_O34.32	Maternal care for cervical incompetence, second trimester	4.36
	D_O09.212	Supervision of pregnancy with history of pre-term labor, second trimester	3.95
	D_O10.912	Unspecified pre-existing hypertension complicating pregnancy, second trimester	3.76
	M_409672903.0	Magnesium Sulfate, Injectable/magnesium sulfate	2.80
	L_3255-7	Fibrinogen Automated	2.49
	D_O41.02X0	Oligohydramnios, second trimester, not applicable or unspecified	2.12

### Limitations

While our study supports the importance EHR data in the prediction of preterm delivery, it highlighted several limitations with this method. The accuracy of selection of the appropriate diagnosis or procedure codes is questioned. Similarly, in order for the laboratory values to make sense, they need to be stratified into positive or negative values. A more clinically relevant prediction model will be able to combine the risk factors and produce a risk index.

## Conclusion

In this work, we used a large dataset from electronic health records to identify risk factors of preterm delivery based on two models namely Logistic Regression and Random Forest. The obtained factors overlap to a large extend between both models. We explored the progression of these risk factors across different stages of the pregnancy. We performed the association analysis at three time gestational ages (0, 12 and 24 weeks). Risk factors before and in early pregnancy related to chronic condition and general predisposition such as hypertension, diabetes and anatomy. Risk factors at advanced stage of pregnancy are mostly related to the current pregnancy and reflect acute condition such as infections, complications in blood. Most of the identified factors could be confirmed from the literature. The results from this experiment are easily reproducible to other diseases. Some risk factors can change over time with the change in the society such as lifestyle, income level etc. Re-evaluating the risk factors will be easy using electronic health records. As future work, we plan to extend this analysis by adding other modalities (microbiology, physiological measurements) and test results of lab order to better refine the obtained results.

## Data Availability

The data is part of Cerner Health Facts. It is protected by copyright and obtained from Cerner through a Data Use Agreement.

## Footnotes

1 https://scikit-learn.org

2 https://www.cdc.gov/reproductivehealth/maternalinfanthealth/pretermbirth.htm

3 https://github.com/bsabri/ptb_ehr
